# A Web-Based Time-Use Application to Assess Diet and Movement Behavior in Asian Schoolchildren: Development and Usability Study of My E-Diary for Activities and Lifestyle (MEDAL)

**DOI:** 10.2196/25794

**Published:** 2021-06-09

**Authors:** Airu Chia, Muhammad Naeem Jia Sheng Chew, Sarah Yi Xuan Tan, Mei Jun Chan, Marjorelee T Colega, Jia Ying Toh, Padmapriya Natarajan, Carla Lança, Lynette P Shek, Seang-Mei Saw, Falk Müller-Riemenschneider, Mary Foong-Fong Chong

**Affiliations:** 1 Saw Swee Hock School of Public Health National University of Singapore and National University Health System Singapore Singapore; 2 Singapore Institute for Clinical Sciences Agency for Science, Technology and Research Singapore Singapore; 3 Department of Obstetrics and Gynaecology Yong Loo Lin School of Medicine, National University of Singapore Singapore Singapore; 4 Singapore Eye Research Institute Singapore National Eye Centre Singapore Singapore; 5 Department of Pediatrics Yong Loo Lin School of Medicine, National University of Singapore Singapore Singapore; 6 Duke-NUS Medical School Singapore Singapore

**Keywords:** time use, web-based, diet, movement behaviors, usability, schoolchildren

## Abstract

**Background:**

Web-based time-use diaries for schoolchildren are limited, and existing studies focus mostly on capturing physical activities and sedentary behaviors but less comprehensively on dietary behaviors.

**Objective:**

This study aims to describe the development of My E-Diary for Activities and Lifestyle (MEDAL)—a self-administered, web-based time-use application to assess diet and movement behavior—and to evaluate its usability in schoolchildren in Singapore.

**Methods:**

MEDAL was developed through formative research and an iterative user-centric design approach involving small groups of schoolchildren (ranging from n=5 to n=15, aged 7-13 years). To test the usability, children aged 10-11 years were recruited from 2 primary schools in Singapore to complete MEDAL for 2 weekdays and 2 weekend days and complete a 10-item usability questionnaire.

**Results:**

The development process revealed that younger children (aged <9 years) were less able to complete MEDAL independently. Of the 204 participants (118/204, 57.8% boys, and 31/201, 15.4% overweight) in the usability study, 57.8% (118/204) completed 3 to 4 days of recording, whereas the rest recorded for 2 days or less. The median time taken to complete MEDAL was 14.2 minutes per day. The majority of participants agreed that instructions were clear (193/203, 95.1%), that MEDAL was easy to use (173/203, 85.2%), that they liked the application (172/202, 85.1%), and that they preferred recording their activities on the web than on paper (167/202, 82.7%). Among all the factors evaluated, recording for 4 days was the least satisfactory component reported. Compared with boys, girls reported better recall ability and agreed that the time spent on completing 1-day entry was appropriate.

**Conclusions:**

MEDAL appears to be a feasible application to capture diet and movement behaviors in children aged 10-12 years, particularly in the Asian context. Some gender differences in usability performance were observed, but the majority of the participants had a positive experience using MEDAL. The validation of the data collected through the application is in progress.

## Introduction

### Background

The prevalence of obesity in children and adolescents has increased in recent decades, and this rising trend has surged in parts of Asia [1]. In addition to short-term health consequences, overweight and obese children have a greater risk of adverse psychosocial health conditions and earlier onset of noncommunicable diseases [2]. Understanding factors contributing to obesity risk in children, such as physical inactivity and poor diet, is critical to prevent the accelerating burden of chronic diseases in later life.

However, there is a lack of good quality and timely data on schoolchildren, particularly those aged 10-14 years [3]. Parents may help to report the health behaviors of their children at home but often are not fully aware of their activities out of home, especially when children are increasingly independent in their food and activity choices at this age [4]. Although questionnaires completed by children are valuable tools to collect health behavioral data, they have the following limitations: (1) they often focus on a single health behavior; (2) they typically involve long-term recall, which is cognitively demanding for children (eg, examine activities during the past 7 days or last month) [5,6]; and (3) they rarely capture emerging health behaviors, such as nonscreen time sedentary behaviors, light-intensity physical activities, outdoor time, and tasks done concurrently (eg, screen time and eating) [7,8]. Although objective methods of measuring health behaviors are increasingly being used, they do not provide information on the type of activity or food consumed [9,10]. To address these limitations, a growing number of studies have used the time-use approach in children to collect information on health behaviors [11].

Time-use diaries prompt users to provide an account of a series of activities in a 24-hour day through previous day recalls [11]. Children are not cognitively developed to recall well before 8 years of age, but by the age of 10 years, they have the independent ability to provide accurate reports [12,13]. In addition, they allow the simultaneous capture of multiple energy balance–related behaviors. Most existing studies rely on paper-based diaries [11], whereas recent studies have developed software-based diaries [14-17], which have been demonstrated to engage children more effectively. However, to our knowledge, these studies focus mostly on capturing physical activities and sedentary behaviors but less comprehensively on dietary behaviors; for example, types of food consumed [14,17] and food portions [15,16] were not collected. Furthermore, there is a lack of application containing databases of Asian foods and common activities of children within the Asian context, such as tuition, also known as shadow education, which is prominent in East Asian countries [18]. In addition, the location of activities has been less studied, and no study to date has collected data on simultaneous activities (eg, eating while studying) in children aged 10-14 years, which is valuable for a better understanding of the activity contexts in children [19].

### Objectives

To bridge these gaps, we developed a self-administered, web-based time-use application to assess diet and movement behaviors in schoolchildren: My E-Diary for Activities and Lifestyle (MEDAL). This study aims to describe the development of MEDAL and evaluate its usability among schoolchildren in Singapore.

## Methods

**Figure 1:**
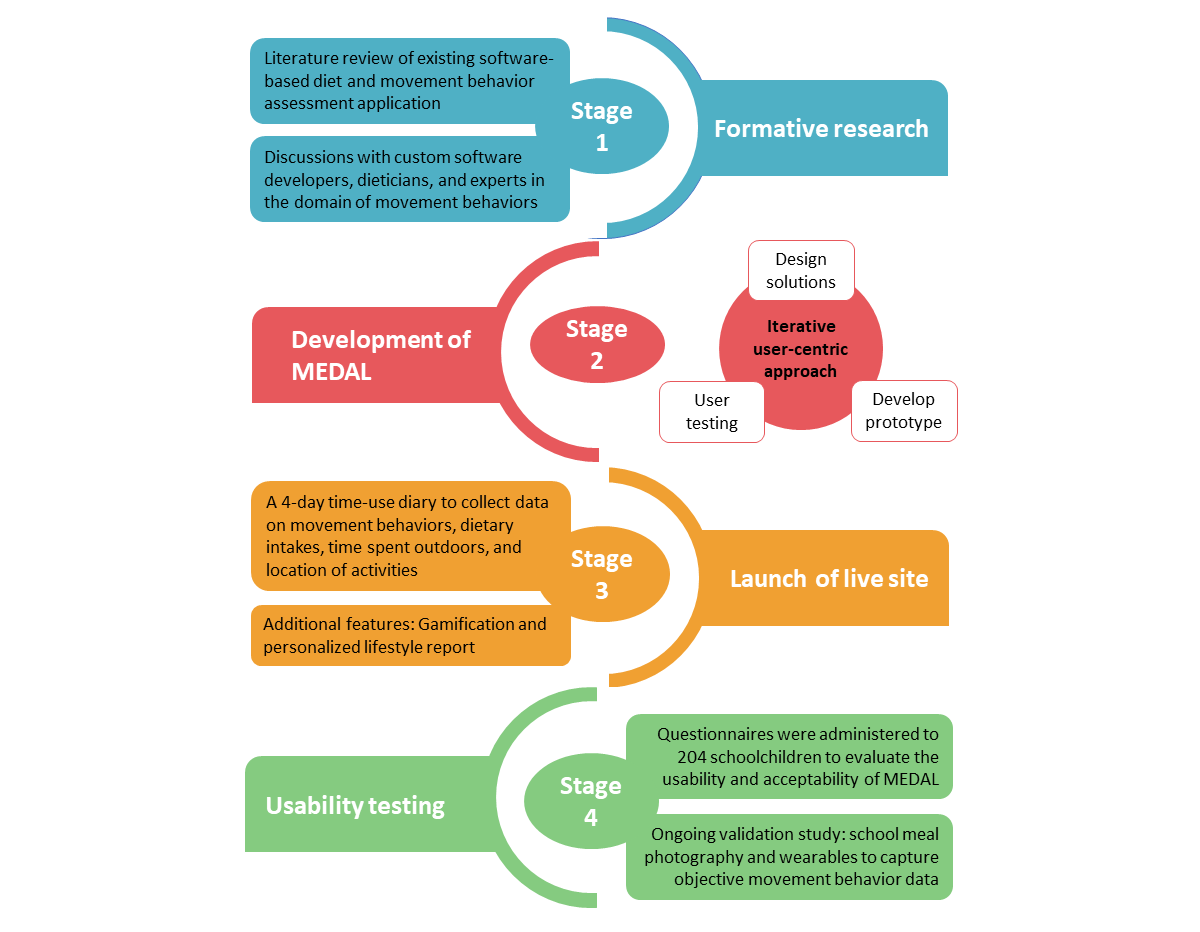
Flowchart illustrating the development process of My E-Diary for Activities and Lifestyle (MEDAL).

The development of MEDAL was a multistage, user-centric process (Figure 1).

### Stage 1: Formative Research

Several data sources were reviewed to identify the elements and requirements for developing a self-administered, web-based application for assessing diet and movement behaviors in schoolchildren. Both electronic and hand-searched strategies were adopted to identify existing software-based diet and physical activity assessment tools for schoolchildren. MyDailyMoves in the Netherlands [17], WebCAAFE in Brazil [16], Synchronised Nutrition and Activity Program in England [15], and Multimedia Activity Recall for Children and Adolescents in Australia [14] were evaluated. We also held interviews and meetings with experts from various knowledge domains, including custom software developers, experienced educators, and dieticians. The key points emanating were (1) application should be simple, easy to navigate, and take no more than 20 minutes per day to complete; (2) use of appealing visuals to engage children, and they prefer images to text and clicking instead of typing; and (3) enable children to report activities sequentially from wake-to-bed time (analogous to a time-use diary), so as to enhance recall—this method of *behavior chaining* has been shown to help children remember their activities [15]—and (4) complete the application at the end of each day to minimize omissions and intrusions [14,20]; children’s accuracy in reporting has been shown to improve with shorter retention intervals (ie, time between event occurrence and reporting) [21].

### Stage 2: Iterative User-Centric Development Process

On the basis of the formative research in stage 1, we adopted an iterative stepwise refinement approach to design, develop, and test features of the application [22]. User testing was conducted with children using methods such as paper-based simulations, audio clips, and mock-ups (ie, static wireframes) to understand the children’s preferences for the interface and their comprehension of the task. At the end of user testing, user feedback was evaluated, and necessary improvements and amendments were conveyed to the software developers to inform the next development cycle. Multimedia Appendix 1 summarizes the key feedback and changes made during each development phase. Small groups of schoolchildren (aged 7-13 years), ranging from n=5 to n=15, were involved in all development stages to ensure that design decisions were tailored to their needs until the full functional system was launched. The sample sizes for each stage of user testing were adequate, as studies have shown that 80% of the usability problems were found with the first 5 users [23], and the diminishing return of uncovered problems was apparent when the sample size increased [24]. The key takeaways from the development phase were as follows: (1) recording should be limited to 4 days (similar to other diet and activity monitoring tools), as children found it tedious to record for 7 days; (2) prompts should be included to aid recall; (3) child-appealing visuals and intuitive designs should be used to ease navigation and motivate completion; and (4) younger children (<9 years) may need more assistance to complete MEDAL independently.

### Stage 3: Launch of the Live Site

#### Technical Specifications

Multimedia Appendix 2 presents the major functionalities of MEDAL. MEDAL is a self-administered, web-based time-use application that takes users through the completion of a 4-day diary to collect data on movement behaviors, dietary intakes, time spent outdoors, and the location of these activities. It is written using the Yii Framework (version 1.0), PHP (version 5.6) as the application programming interface, Angular 2 (version 2) as the front end, MySQL (version 8) as the database, and hosted by the National University of Singapore. It is accessible on browsers such as Google Chrome, Microsoft Edge, Firefox, and Safari on devices such as tablets, laptops, and desktops.

#### Log-in and Registration

User-specific accounts are created by research administrators through the backend portal, and users can access MEDAL by logging on with a username and password given to them. Information on the date of birth and sex of the user is collected during the first log-in. MEDAL allows users to have the flexibility to record on any 4 days. Users can select if they are recording for the current day or the day before. The recall period is limited to the day before to minimize the retention interval and increase the likelihood of data validity [21].

#### Recording of Activities and Diet

##### Activities

Users are asked about the time they slept the night before and their wake-up time. Following this, users are asked to record their activities sequentially, with the end time of one activity leading to the start of the next, until the end of the day is reached (ie, bedtime). They can choose from 22 activities grouped into 6 broad categories: (1) shower or wash up, (2) eat and drink, (3) traveling, (4) sitting activities, (5) active activities, and (6) nap/sleep. For example, the traveling category included activities such as *walk*, *cycle*, *car/taxi*, and *bus/train*; sitting activities category included *reading/doing homework, hand phone/tablet, TV, video games, computer/laptop, board/card games, sit and chat,* and *tuition/music lesson*; and active activities included *run/jog, cycle/skate/scoot, ball games, martial arts, dance, neighborhood play, and water activities*. This list of activities was compiled from existing questionnaires examining the indoor and outdoor activities of schoolchildren [25]. Common activities listed in 7-day activity diaries completed by 200 children of a local multiethnic birth cohort study (Growing Up in Singapore Towards healthy Outcomes) [26] were also considered (Cheryl Zhang, MPH, unpublished data, May 2018). These activities were further grouped to reduce the number of response options (eg, basketball, handball, and football are collectively known as *ball games*), as a previous report showed that providing a large number of options for children was associated with poorer quality of data collected [27]. Children can specify activities that are not listed by typing in a free-text box labeled *other*.

When an *active activity* is selected, users will be questioned further about its intensity: “How tiring was the activity?” To aid children in conceptualizing activity intensity, 3 emoticons with captions illustrating different degrees of physical exertion are presented: (1) Just a little—you can sing and talk during the activity, (2) Quite tiring—you can talk but cannot sing during the activity, and (3) Very tiring—you cannot say more than a few words without pausing. Energy expended at each activity can be determined by multiplying the duration of the activity and its corresponding metabolic equivalent task, drawn from the Youth Compendium of Physical Activities [28].

##### Diet

When the category *eat and drink* is chosen, users will be asked to select the food and drink they consumed from 88 food items (icons) organized under 13 main food and drink groups (Multimedia Appendix 3). When necessary, items were placed in multiple food groups so as to enhance user accessibility (eg, French fries was located in the *noodle/pasta/potatoes* group and in the *fast food* group). Besides browsing the food and drink groups, users can perform a text search to display the food items as typed. The embedded fuzzy search feature allows items to be searched and displayed even when the food names are misspelled, thus greatly enhancing the ease of use of MEDAL for children. The list of food and drinks was compiled from commonly consumed foods identified from 3-day diet records of 360 local children of the Growing Up in Singapore Towards healthy Outcomes cohort [29] as well as discussions with experts in the field of dietary assessment. Users were also asked to choose the portion sizes of food that best corresponded to the amount they consumed from 4 pictorial options (shown simultaneously) per food item. A total of 4 pictorial options were provided, as this was found to be the optimal number of options for children to select from [20]. Moreover, the even-numbered options limit the middle option from being chosen out of convenience [30]. Simultaneous presentation of food images depicting increasing portion sizes is preferred over the sequential presentation for children to quantify their food intake more accurately [31] and within a shorter period [32]. For items that are not listed, users can type into the *other* textbox and indicate the amount consumed from the 4 options provided: half a portion, 1 portion, one-and-a-half portion, or 2 portions. Prompts for commonly forgotten foods “Did you forget to select any fruits, drinks, dessert, or supplements you have eaten?” were provided after each meal entry. Users also have the option to review and edit the food and drink items entered before recording the next activity.

##### Additional Features

MEDAL requests users to indicate activities that were performed simultaneously (eg, watching TV while having their meals), which may not be achievable in existing assessment tools [14-16]. Users were also prompted to enter the location of their reported activities (ie, home, school, student care, or others) and if they were done indoors or outdoors. Inquiry on location helps children to contextualize and recall the activities they did [15,20]. Following the selection of an activity, children were asked to record the end time of the activity. The recorded end time should be at least 5 minutes from the start time. The minimum time frame of 5 minutes was designed to allow children to record short bouts of activity [14]. To check for activities that may be missed out, prompts such as “That’s an odd timing, are you sure you ended this at [time]?” will be provided if users recorded an activity that lasted for more than 3 hours. MEDAL automatically saves the user’s responses and allows them to log in at any time of the day to continue recording or review and edit their activities if they are not able to complete in one sitting.

#### Gamification

Several game design elements were implemented to motivate children to complete MEDAL. Users can personalize the background interface and select their choice of avatar. The chosen avatar provides instructions and prompts in the form of visual texts and auditory dialogs, which serve to alert the user to action (ie, more reactive) and enhance user immersion in the MEDAL task [33].

Each completed day of recording rewards users with a gardening tool to nurture their virtual plant. At the end of 4 complete days of recording, a randomly assigned mystery fruit will be revealed to the user. These visual representations of achievements directly communicate the success or completion of the task and serve to evoke feelings of competence and efficiency in children [34]. The addition of the mystery element and randomization further aims to spark curiosity in children and motivate them to complete the task [35].

#### Personalized Lifestyle Report

On completing 4 days of recording, users will instantly receive a personalized lifestyle report. The report provides information on 4 components of movement behaviors—physical activity duration, outdoor duration, screen viewing duration, and sleeping duration—and evaluates the intake of 5 food groups—fruits, vegetables, whole grains, sugar-sweetened beverages, and dairy and dairy products. The mean durations or servings per day (ie, daily estimates) were calculated based on standardized algorithms and compared with the recommended guidelines [36-38]. Specific messages were delivered to users for each component based on the categories they belong: (1) met recommended guidelines, for example, “Terrific! Super-duper proud of you! You should continue to spend less time on the screen”; (2) nearly meeting guidelines, for example, “Almost there! Aim to spend less time on the screen. Keep going!”; or (3) did not meet guidelines, for example, “You should spend less time on the screen. Aim for less than 2 hours of screen time daily. Persevere! I believe in you!” Messages were gain-framed (ie, focused on attaining a desirable or positive outcome) and presented with graphical illustrations, as evidence from existing reviews and qualitative research has shown that this is effective in motivating children [39].

### Stage 4: Usability Testing

#### Study Population

To test the usability of MEDAL, primary 5–level children (aged 10-11 years) from 2 coeducational government schools in Singapore were invited to participate in the usability study between April and August 2019. Children were eligible if they were able to understand and respond to the assessment in English. Of the 458 invited children from 13 classes (7 classes from school A and 6 classes from school B), 49.8% (228/458) were recruited. The main reasons for nonparticipation were *did not return consent form* (150/458, 32.8%), *parents did not consent* (46/458, 10%), *child was busy or not interested* (20/458, 4.4%), and *child was absent on the day of study* (14/458, 3.1%). The study was approved by the institutional review board of the National University of Singapore (Reference S-18-088). Approval was also obtained from the Ministry of Education, Singapore, to approach schools for participant recruitment (reference RQ17-18(02)B). Informed written consent was obtained from the parents or guardians, and all participants gave verbal assent.

#### Data Collection

Practical sessions were conducted for each class in the schools’ computer laboratories by 2 trained researchers who demonstrated how to log in and navigate the MEDAL application. Participants started their first MEDAL entry using school computers and completed the rest of the day’s entry at home. Participants were instructed to record their diet and activities over 2 specified weekdays and 2 weekend days. They were encouraged to complete the day’s entry at the end of the day (ie, before bedtime) or the day after. The schools’ computer laboratories were opened during recess and after school to allow participants with no access to the internet or computer at home to complete MEDAL. School meal photography during the recess period was also conducted, and devices to capture objective data on movement behaviors (ie, accelerometers) and outdoor time (ie, FitSight watch, which measures light intensity [40]) were provided for validation of data collected through MEDAL.

At the end of the study, participants were instructed to complete a 10-item usability questionnaire based on a 4-point Likert scale (strongly disagree, disagree, agree, and strongly disagree). The questionnaire was adapted from existing usability questionnaires [41,42] and kept short to avoid satisficing responses [43]. Questions were positively worded, direct, and specific to tailor to the children’s cognitive and communicative capacities [27]. The key themes covered by the questionnaire included clarity of instructions, motivation to continue recording, ability to recall activities and food, recording duration, and overall experience of using MEDAL. Participants were also allowed to provide comments and suggestions at the end of the questionnaire.

Information on participants’ sex and age was collected from MEDAL. The time taken for participants to complete MEDAL each day was calculated as the duration of the first and last entry input (ie, bedtime), summed and averaged over the number of recording days. Height and weight were measured biannually in schools, and the most recent records were obtained. Underweight (<5th percentile), normal weight (5th to 90th percentile), and overweight (>90th percentile) were classified according to the age- and sex-specific BMI reference of the local population [44]. Parents were asked to indicate their child’s access to computers and the internet at home (yes or no) in the consent form.

#### Statistical Analyses

Participants’ characteristics were summarized according to sex, and comparisons were assessed by the Fisher exact test (categorical) and Wilcoxon Mann-Whitney test (continuous). Differences in usability attributes between sexes were examined using the Fisher exact test. The usability questionnaire responses *agree* and *strongly agree* as well as *disagree* and *strongly disagree* were merged into 2 categories, *agree* and *disagree*, before analysis. The method has been shown to be more helpful in the description of Likert scale data [45]. All statistical analyses were performed using Stata 14 (StataCorp LP). Statistical significance was set at *P*<.05.

## Results

### Characteristics of Participants

**Figure 2:**
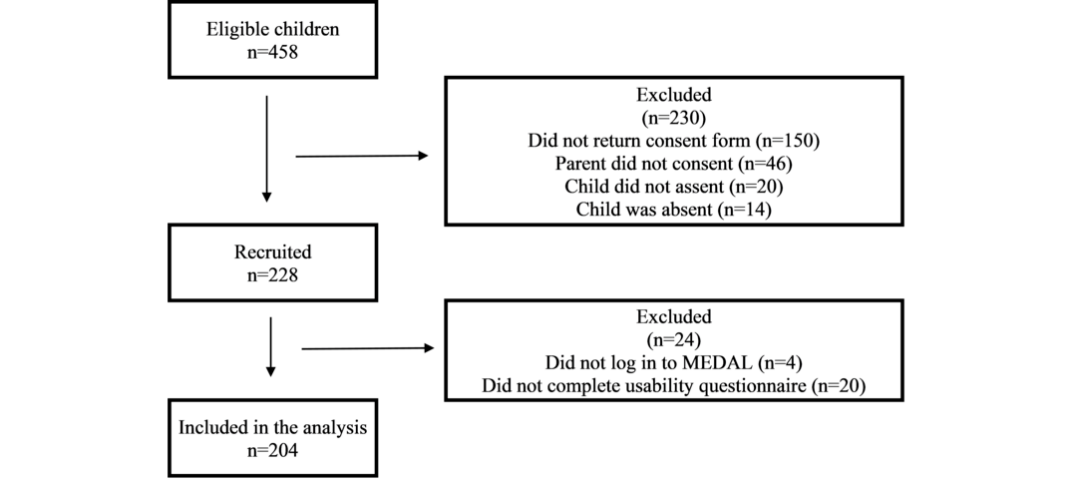
Flowchart of participants. MEDAL: My E-Diary for Activities and Lifestyle.

Of the 228 participants, 204 logged on to MEDAL and completed the usability questionnaire and were included in the analysis (Figure 2). The cohort comprised 57.8% (118/204) boys, with a median age of 10.9 (25th percentile: 10.6 and 75th percentile: 11.2) years, and 15.4% (31/201) were overweight (Table 1). The median time taken to complete MEDAL was 14.2 minutes per day, and the proportion who completed 3-4 days of recording, 1-2 days of recording, and <1 day of recording were 57.8% (118/204), 20.1% (41/204), and 22.1% (45/204), respectively. Compared with boys, girls spent more time completing MEDAL (median 15 min per day vs 13 min per day; *P*=.03) and were more likely to complete 3-4 days of recording (*P*=.01).

**Table 1:** Characteristics of participants summarized by sex (N=204).

Characteristics of participants			All (N=204)		Girls (n=86)		Boys (n=118)		*P* value^a^
**School, n (%)**									.07
	School A	141 (69.1)		53 (61.6)		88 (74.6)			
	School B	63 (30.9)		33 (38.4)		30 (25.4)			
Age (years), median (25th-75th percentile)			10.9 (10.6-11.2)		11.0 (10.8-11.2)		10.9 (10.6-11.1)		.03
**Weight status, n (%)**									.41
	Underweight	25 (12.4)		12 (14)		13 (11.3)			
	Normal weight	145 (72.1)		64 (74.4)		81 (70.4)			
	Overweight	31 (15.4)		10 (11.6)		21 (18.3)			
**Access to the internet and computer at home, n (%)**									.99
	Yes	170 (89.5)		73 (90.1)		97 (89)			
	No	20 (10.5)		8 (9.9)		12 (11)			
**Days completed, n (%)**									.01
	<1 day	45 (22.1)		12 (14)		33 (28)			
	1-2 days	41 (20.1)		13 (15.1)		28 (23.7)			
	3-4 days	118 (57.8)		61 (70.9)		57 (48.3)			
Average time taken to complete in minutes per day, median (25th-75th percentile)			14.2 (10.4-19.0)		15.0 (11.1-20.3)		13.1 (10.1-17.6)		.03

^a^*P* values were assessed by the Fisher exact test (categorical) and Wilcoxon Mann-Whitney test (continuous). There were missing data for weight status (n=3) and access to the computer and internet at home (n=14).

### Usability of MEDAL

When assessing the usability of MEDAL, the majority (193/203, 95.1%) of participants agreed that instructions were clear, that MEDAL was easy to use (173/203, 85.2%), that they (172/202, 85.1%) liked the application, and that they preferred recording their activities on the web than on paper (167/202, 82.7%; Table 2). Recording for 4 days and ability to recall events were reported to be the least desirable among other factors (146/203, 71.9% and 154/203, 75.9%, respectively). Overall, the percentage agreement for the 10 questions ranged from 71.9% (146/203) to 95.1% (193/203).

**Table 2:** My E-Diary for Activities and Lifestyle (MEDAL) usability responses according to boys and girls.

Survey questions	Agree, n (%)^a^				*P* value^b^
	All (n=204)	Girls (n=86)	Boys (n=118)		
1. I understood the instructions for MEDAL^c^ clearly	193 (95.1)	83 (96.5)	110 (94)	.52	
2. It was easy to use and I do not require further instructions	173 (85.2)	76 (89.4)	97 (82.2)	.17	
3. Earning tools to grow my plant makes me want to continue the game	159 (78.7)	75 (88.2)	84 (71.8)	.005	
4. I was able to remember all the activities I did	154 (75.9)	70 (82.3)	84 (71.2)	.07	
5. I was able to remember all the food and drinks I had	165 (81.2)	75 (87.2)	90 (76.9)	.07	
6. The time I took to complete one day’s entry was just right	149 (75.6)	71 (83.5)	78 (69.6)	.03	
7. Recording for 4 days was just right	146 (71.9)	66 (77.7)	80 (67.8)	.15	
8. I prefer recording my activities online than using paper	167 (82.7)	73 (85.9)	94 (80.3)	.35	
9. I would play the game again	162 (79.8)	73 (85.9)	89 (75.4)	.08	
10. Overall, I liked the game	172 (85.1)	76 (89.4)	96 (82.1)	.17	

^a^Values shown are the sums of “Strongly agree” and “Agree.”

^b^*P* values were assessed by Cochran-Mantel-Haenszel test. There were missing data for question 1 (n=1), question 2 (n=1), question 3 (n=2), question 4 (n=1), question 5 (n=1), question 6 (n=7), question 7 (n=1), question 8 (n=2), question 9 (n=1), question 10 (n=2).

^c^MEDAL: My E-Diary for Activities and Lifestyle.

Compared with boys, girls were more motivated by the gamification to complete recording on MEDAL (75/85, 88.2% vs 84/117, 71.8%; *P*=.005) and agreed that the time spent on completing 1-day entry was appropriate (71/85, 83.5% vs 78/112, 69.6%; *P*=.03; Table 2). Compared with boys, a higher proportion of girls reported being able to recall all the activities they did (84/118, 71.2% vs 70/85, 82.3%; *P*=.07) and food and drinks they consumed (90/117, 76.9% vs 75/86, 87.2%; *P*=.07).

### Open-ended Suggestions

A total of 23 participants provided open-ended suggestions. Participants (n=8) who recorded MEDAL for ≤2 days preferred recording on paper rather than on the web (Textbox 1). It was also reported that there was insufficient time to complete MEDAL. For participants (n=15) who completed 3 or 4 days of recording, there were suggestions to include more food and activity options and to have the ability to edit timings of activities (n=3 girls). Participants also proposed to allow recording on MEDAL for more than 4 days (n=3 boys) and modifying the game design, such as inclusion of more avatars and games and wanting a reward that is different from a plant.

**Suggestions provided by 23 participants.**

**Participants Who Recorded for ≤2 Days (n=8)**

- Prefer recording on paper rather than on the web
  “We could use paper to do rather than online” [Girl]
  “I do not want the activity online but in paper” [Boy]
  “To use paper than online is easy. I don’t know” [Boy]
- Insufficient time to complete MEDAL
  “This program is very interesting but a bit troublesome as I don’t have much time to finish the online diary” [Girl]
- Include more options
  “More options. Like more activities.” [Boy]
  “Study in school” [Boy]

**Participants Who Recorded for 3-4 Days (n=15)**

- Include more options
  “Include more options for us to choose” [Girl]
  “Have more food options” [Boy]
  “Sometimes we just do nothing, so please add a button saying ‘nothing’” [Boy]
- Ability to edit timing of activity
  “Delete stuff [cause (sic) I accidentally write what time I sleep wrongly but could not change]” [Girl]
  “During the game, when putting in the timing slots, when I put some numbers in wrong it is impossible to change” [Girl]
  “For example, if you forgot what you did but you suddenly remembered you can change what you wrote” [Girl]
- Allow recording on MEDAL for more than 4 days
  “I would ask them if they wanted to do more” [Boy]
  “Make the days longer” [Boy]
  “The ability to do the entries for the days even further behind” [Boy]
- Modifying the game design
  “Put more characters!” [Girl]
  “I would change the reward given.” [Girl]
  “...to do more and they could get another plant” [Boy]
  “More games” [Boy]
- Allow recording of sleep past midnight
  “Make time for 12 am when I sleep” [Boy]

## Discussion

### Principal Findings

In this study, we described the development and evaluated the usability of MEDAL—a 4-day web-based time-use application to assess diet and movement behavior in schoolchildren in Singapore. Approximately 60% (118/204) of the participants completed more than 3 days of recording, whereas the rest recorded for 2 days or less. The majority of the participants reported having a positive experience using MEDAL. Compared with boys, girls were reported to be more motivated by gamification, had better reported recall ability, and agreed that the time spent on completing 1-day entry was appropriate.

During the development process of MEDAL, we observed that the completion rate of MEDAL was significantly lower in younger children (aged 8-9 years) than in older children (aged 11-12 years). Younger children aged <9 years may need more assistance to complete MEDAL independently; thus, MEDAL is more suitable for children aged ≥10 years.

Response rates for multiday diaries have generally been an issue among children [46]. It is perceived as tedious to report for ≥2 days, especially when activity patterns were more or less similar on school days. Respondents may also report fewer activities on subsequent days as a means of decreasing the level of burden [47]. Indeed, in our study, we found similar issues with response rates, such that only 57.8% (118/204) of the participants completed 3-4 days of recording. Recording for 4 days was also the least satisfactory component reported in the usability assessment. To strike a balance between the reliability of data and minimizing respondent burden, the collection of 2 diary days for a large study (ie, 1 weekday and 1 weekend day) or 3 days for smaller-scale investigations (ie, 1 day from Monday to Thursday, Friday, and 1 weekend day) has been deemed adequate to obtain representative time-use data in children [14,46]. Large sample sizes may level out individual behavioral misclassifications that result from the short assessment period and would consequently give accurate population-level estimates [48]. For the next version of MEDAL, we plan to provide the flexibility to record between 1 and 7 days and optimize the application for mobile devices on both Android and iOS platforms to cater to the needs of different projects, and users can access the website on the go.

We observed that participants who recorded for <2 days were less likely to have access to a computer and the internet at home (*P*=.02, results not shown), and qualitative data showed that they preferred recording on paper rather than on the web. Participants (20/190,10.5%) with no access to the computer and internet at home may be less tech-savvy, thus exhibiting lower usability performance [16] and greater preference for print than digital. In the next version of MEDAL, we plan to integrate a frequently asked questions section to assist participants, especially those with lower computer knowledge and skills, to troubleshoot their problems and enable them to navigate the site with ease.

Existing studies have shown that recall ability is a common difficulty reported by children [14,16], although girls tend to outperform boys in episodic memory tasks (eg, everyday events) [49]. This is in line with our findings, where we observed trends toward greater reported recall ability in girls than in boys. Participants suggested including more food and drink and activity options and pictures (eg, household chores and shopping). This feedback will be incorporated into the next version of MEDAL, as images will be useful to serve as visual memory prompts to enhance recall in children.

Studies have shown that boys have lower attentional performance and poorer inhibitory control but perform faster than girls [50]. Consistent with these findings, we found that girls spent more time completing MEDAL (median 15 min per day vs 13 min per day), but they were more agreeable to the time spent on MEDAL compared with boys. Generally, the actual average time spent on MEDAL was comparable with similar questionnaires completed by children [15,16], which is sufficiently short to hold the users’ attention [20,51].

With regard to gamification, existing studies did not include this element [14-17]. In our study, girls appeared to be more motivated by the virtual gardening game to complete MEDAL recording than boys. Some boys have suggested integrating more games and that they wish to do more so that they can receive more rewards. Introducing more games may distract users and steer them away from recording their daily activities attentively [52]. However, as most of the participants reported a positive evaluation for the in-built game, we plan to revise and accelerate the game progression rewards in the next version of MEDAL so that users are able to receive rewards more frequently. Users will be rewarded with gardening tools as they record their activities, and a mystery fruit will be revealed after each completed day of recording. On the basis of the feedback from the participants, we will also include more avatars and backgrounds for users to choose and personalize in the next version of MEDAL.

### Strengths and Limitations

MEDAL is a novel and engaging application for schoolchildren aged ≥10 years. It includes a range of Asian food options that cater to the key ethnic groups in Asia and captures data on food portions, which allow us to have a detailed assessment of energy and nutrient intakes. This is useful, as evidence suggests that portion sizes among children are increasingly poorly regulated [53]. In addition to assessing dietary intake and movement behaviors, MEDAL also examines the location of activities and tasks performed concurrently to allow a better understanding of the context of the activities and enable interventions to be more targeted. The usability evaluation of MEDAL, including user testing during the development phase, involves several methodological strategies (eg, direct observation, interview, Likert scale questions, and open-ended feedback), which allowed us to obtain rigorous and detailed qualitative and quantitative data.

However, there are some limitations to this study. First, we did not have information on children’s academic ability. However, our usability questionnaire showed that 95.1% (193/203) of the participants understood the instructions on MEDAL, which was not unexpected, considering that English is the main language of instruction in Singapore and primary education is compulsory. However, we recognize that the cognitive abilities to recall events, capability to concentrate, and comprehension of questions may have an impact on usability testing [16] and acknowledge that children with poorer cognitive ability may experience some challenges in using MEDAL. Currently, users are only able to edit types of activities but not the timing of the activities. In addition, the current application prevents users from recording beyond 12 midnight. As late bedtimes have become pervasive in children [54], the ability to record activities beyond 12 midnight, along with the function to modify timing, will be made available in the next version of MEDAL. In addition, to cater to both observational and interventional study designs, we plan to incorporate the flexibility to hide the lifestyle report so that it will not induce behavioral change in participants of observational studies.

### Conclusions

In conclusion, we developed MEDAL, which appears to be a feasible web-based time-use application for children aged 10-12 years and potentially useful for children above this age group. We observed some gender differences in usability performance, but the majority of the participants had a positive experience using MEDAL. The validity and reliability of MEDAL will be examined next, and the results of this study will be used to inform the next version of MEDAL to customize MEDAL for children in other parts of Asia, which includes translation to their native language and adaptation of application content to their local context.

## Appendix

Multimedia Appendix 1 Development process of My E-Diary for Activities and Lifestyle (MEDAL).

Multimedia Appendix 2 Functionality of My E-Diary for Activities and Lifestyle (MEDAL).

Multimedia Appendix 3 Food items under the eat and drink category.

## Abbreviations

MEDAL: My E-Diary for Activities and Lifestyle
