# Supplementary material for: A Web-Based Time-Use Application to Assess Diet and Movement Behavior in Asian Schoolchildren: Development and Usability Study of My E-Diary for Activities and Lifestyle (MEDAL)
Source: J Med Internet Res. 2021 Jun 9;23(6):e25794. doi: 10.2196/25794 (PMC8262598; doi:10.2196/25794)
Supplement: Multimedia Appendix 1 [file jmir_v23i6e25794_app1.doc]

**Multimedia Appendix 1**: Development process of MEDAL

| Development process | Participants characteristics | Key feedback | Improvements made |
| --- | --- | --- | --- |
| **Hardcopy time-use diary**  A 7-day paper diary was given to children to record as the day goes by and use these notes to complete the online diary at the end of the day. | *n* = 8  3 M, 5 F  9 – 12 years | - Instructions were hard to understand | - Simplified instructions and used illustration to convey complex concepts |
|  |  | - Tend to forget fruits, desserts, snacks, and drinks | - Included prompt “Did you forget to select any fruits, drinks…” |
|  |  | - Typically, 2-3 days were recorded for. Children found it tedious to complete for 7 days | - Shortened recording to 2 weekdays and 2 weekend days |
| **Simulation of MEDAL on paper**  Layouts and designs were shown to the children and interviews were conducted to understand their preferences for the usability features. | *n* = 14  5 M, 9 F  7 – 13 years | - Prefer personalising user account | - User can choose their character and background |
|  |  | - Favour a lively and engaging web design | - Added voiceover and animations e.g. ‘talking’ character |
|  |  | - Input of timing is confusing | - Simplified keypad allows clicking and AM PM toggle to input time |
|  |  | - Forgot to report some activities | - Prompt if activity lasts > 3h “That’s an odd timing, are you sure…” |
|  |  | - Images help users to recall what they did or ate | - All response options were represented by images and a short caption |
|  |  | - Lazy to recall and tend to choose “I don’t know” | - Removed “I don’t know” option to boost memory recall |
|  |  | - Lack of motivation to record for 4 days | - Incorporated plant growing game to entice users to continue recording |
|  |  | - Not all activities or foods were listed | - Included a free-text box for users to input unlisted food or activities |
| **Understanding of location**  Images of common places were presented and children had to answer “Indoor” or “Outdoor” in the 16-item questionnaire. | *n* = 15  8 M, 8 F  7 – 10 years | A majority of questions were answered correctly by all the children. |  |
| **Voiceover**  Five audio clips were shortlisted for children to indicate their preference for the voiceover. | *n* = 7  3 M, 4 F  7 – 11 years | One of the five audio clips was chosen based on majority votes. |  |
| **Portion size estimation**  Images of foods from smallest to largest portions were presented and children were asked to circle the image which best represents their typical intake. | *n* = 8  6M, 2 F  8 – 11 years | - Captions were not adequate e.g. ‘Dry noodles’ | - Included examples of commonly consumed food   e.g. Dry noodles (e.g. wanton noodles, mee goreng) |
|  |  | - Did not understand abbreviations like ‘dsp’ | - Used ‘pieces’ or ‘slices’ instead |
|  |  | - Did not understand decimals | - Replaced values with words e.g. ‘Half a rice bowl’ |
|  |  | - Portion provided for some items are inadequate | - Included images of larger portion size |
| **MEDAL mock-up (static)**  Children were presented with the mockups (i.e. static wireframe with visual design) and they provided their feedback on the accessibility and usability. | *n* = 5  3M, 2 F  7 – 11 years | - Prefer characters to be domestic animals | - Removed ‘Armadillo’ ‘Ostrich’ and replaced with ‘Cat’ ‘Dog’ |
|  |  | - Background is too dim | - Increased brightness of the background |
|  |  | - Recalling the duration of the activity is tedious | - Asked for end time instead of duration |
|  |  | - Horizontal navigation bar is not intuitive | - Revised to side (vertical) navigation bar to show food categories |
|  |  | - Search box for ‘Eat & Drink’ is not prominent | - Included a placeholder ‘Type to search…’ |
|  |  | - Portion size images are too small | - Enlarged images by reducing the number of images from 5 to 4 per item |
|  |  | - Inadequate description of activity intensity | - Added caption e.g. ‘you can talk but cannot sing during the activity.’ |
| **MEDAL prototype (interactive)**  Children were encouraged to navigate MEDAL independently and observers (one observer per child) watch their click-paths and navigation sequence and note down any usability problems. | *n* = 10  6M, 4 F  10 – 11 years | - Skip questions without answering | - Users are only allowed to proceed when all questions are answered |
|  |  | - Hard to tell if the option was chosen | - Increased contrast between selected option and background |
|  |  | - Some buttons (e.g. AM PM) are not conspicuous | - Coloured differently to be more clearly differentiated |
|  |  | - Portion size images are too small | - Minimize other images on the screen to enlarge portion pictures |
|  |  | - Hard to recall if user record at the end of the day | - User can record as and when and the data is auto-saved |
|  |  | - Predictable game – only one type of fruit reward | - Included four types of mystery fruits randomly assigned to user |
|  |  | - Miscalculation of values in lifestyle report | - Bug fixed and checked against a sample of completed MEDAL entries |
| **Pilot testing**  Children were tasked to complete MEDAL for 3 weekdays and 1 weekend day.  *We have only recruited Primary 3 and Primary 6 children and the other levels were not chosen due to other school commitments during the study period. | *n* = 20  7M, 13 F  8 – 9 years  Primary 3  *n* = 26  13M, 13 F  11 – 12 years  Primary 6 | 21% of children aged 8-9 and 60% aged 11-12 years completed 4 days of recording while the rest completed 1-3 days of MEDAL. | Younger children (<9 years) may need more assistance to complete MEDAL independently. |
