# Supplementary figures and images for: A Web-Based Time-Use Application to Assess Diet and Movement Behavior in Asian Schoolchildren: Development and Usability Study of My E-Diary for Activities and Lifestyle (MEDAL)
Source: J Med Internet Res. 2021 Jun 9;23(6):e25794. doi: 10.2196/25794 (PMC8262598; doi:10.2196/25794)

#
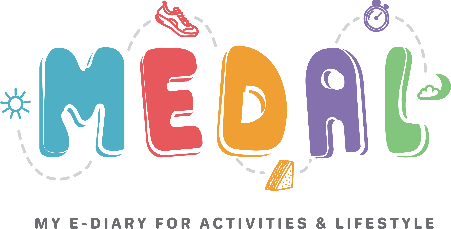
 Multimedia Appendix 2. Functionality of MEDAL­­­


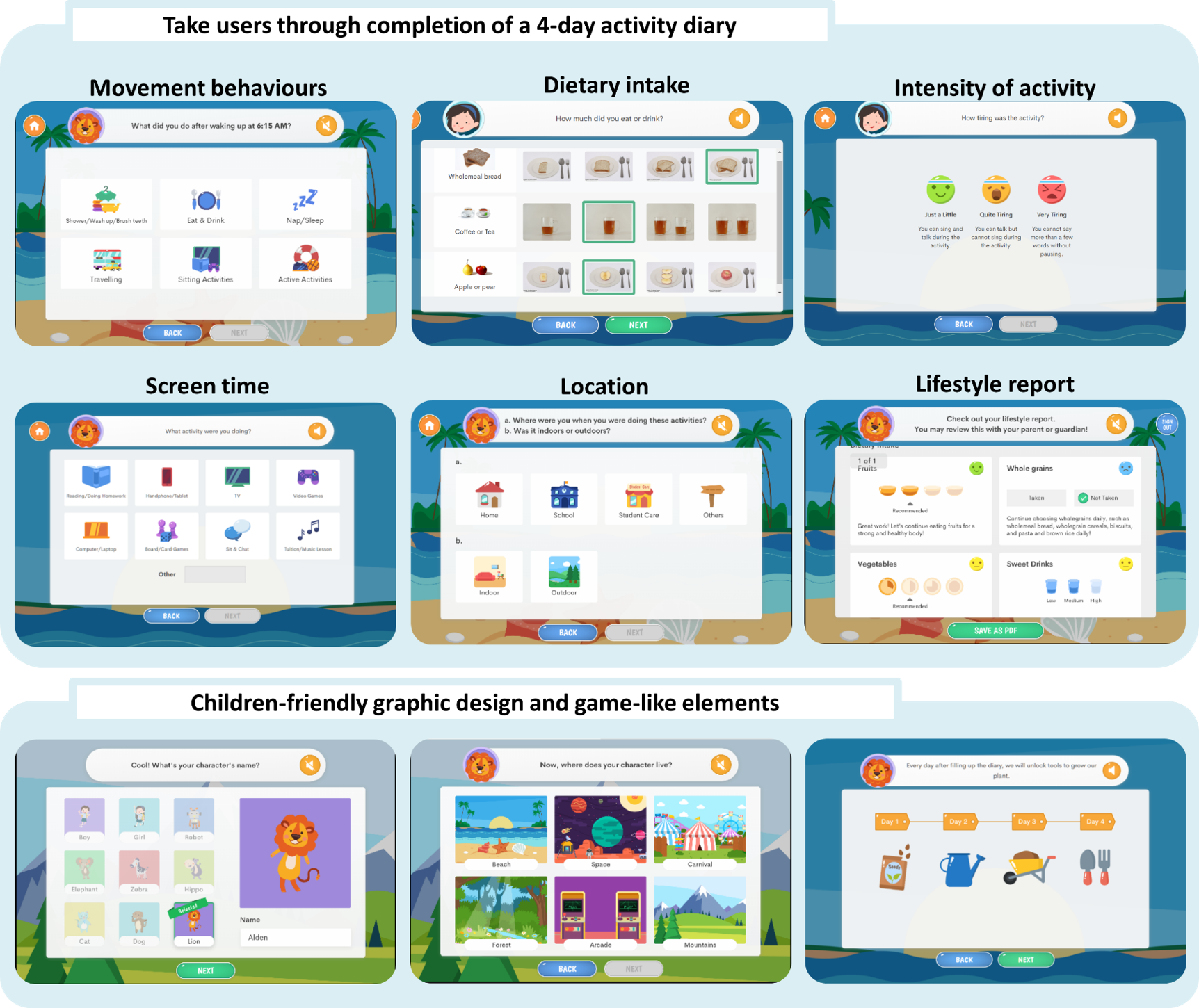

Supplement: Multimedia Appendix 2 [file jmir_v23i6e25794_app2.doc]
