# Supplementary material for: A Web-Based Time-Use Application to Assess Diet and Movement Behavior in Asian Schoolchildren: Development and Usability Study of My E-Diary for Activities and Lifestyle (MEDAL)
Source: J Med Internet Res. 2021 Jun 9;23(6):e25794. doi: 10.2196/25794 (PMC8262598; doi:10.2196/25794)
Supplement: Multimedia Appendix 3 [file jmir_v23i6e25794_app3.doc]

# Multimedia Appendix 3. Food items under the Eat and Drink category

| **Breads, Spreads and Cereals** |  | **Milk, Cheese, and Yogurt** |
| --- | --- | --- |
| White bread |  | Full-fat milk |
| Wholemeal bread |  | Semi-skimmed milk |
| Bread spreads (e.g. butter, jam) |  | Chocolate or strawberry milk |
| Buns or pao |  | Yogurt |
| Breakfast cereals (e.g. Koko Krunch®, Cornflakes) |  | Cheese |
| Roti prata |  | Yogurt drink or milk shake |
| Chapati |  |  |
| Thosai or idli |  | **Eggs and Tofu** |
| Oats |  | Boiled or steamed eggs |
|  |  | Fried or scrambled eggs |
| **Rice and Porridge** |  | Tofu |
| White rice |  |  |
| Brown rice |  | **Curry** |
| Mixed white and brown rice |  | Curry gravy only |
| Fried rice |  | Curry vegetables (e.g. daal, sayur lodeh) |
| Chicken or duck rice |  | Curry meat or fish |
| Nasi lemak |  |  |
| Plain porridge |  | **Meat and Fish** |
| Chicken or fish porridge |  | Stir-fry or steamed chicken |
| Sushi |  | Deep fried chicken |
|  |  | Nuggets or sausages or meatballs |
| **Noddles, Pasta and Potatoes** |  | Stir-fry or steamed meat (e.g. pork, beef or mutton) |
| Dry noodles (e.g. wanton noodles, mee goreng) |  | Deep fried meat |
| Noodles in soup (e.g. beehoon soup, mee soto) |  | Ham or luncheon meat |
| Noodles with gravy (e.g. mee rebus, hor fun) |  | Stir-fry or steamed fish |
| Pasta with sauce (e.g. bolognese, carbonara) |  | Deep fried fish |
| Pasta in soup (e.g. macaroni or alphabet soup) |  | Fishball or fishcake or crabstick |
| Boiled or cooked potatoes (e.g. mashed or sweet potatoes) |  | Curry meat or fish |
| French fries or hash brown |  | Prawn or other shellfish |
| Instant noodles |  | Dimsum (e.g. dumplings, siew mai) |
|  |  |  |
| **Fast food** |  | **Mushroom and Vegetables** |
| Burger |  | Broccoli or cauliflower |
| Pizza |  | Dark green leafy vegetables (e.g. spinach, kailan) |
| French fries or hash brown |  | Light green vegetables (e.g. cabbage, beansprouts) |
|  |  | Yellow, orange, red vegetables (e.g. corn) |
| **Snacks and Desserts** |  | Curry vegetables (e.g. daal, sayur lodeh) |
| Biscuits or cookies |  | Peas and green beans |
| Chips (e.g. potato chips, keropok, murukku) |  | Lentils and other beans (e.g. chickpeas, broad beans) |
| Ice-cream |  | Pumpkin |
| Cakes, kueh kueh or waffles |  | Mushroom |
| Jelly or pudding (e.g. fruit jelly, fruit pudding) |  |  |
| Chocolates |  | **Fruits** |
| Sweets |  | Apple or pear |
| Nuts |  | Banana |
| Pastries (e.g. curry puff, apple pie, crossiant) |  | Grapes or berries (e.g. strawberries, blueberries) |
| Dessert in soup (e.g. green bean soup, ice kachang) |  | Melons (e.g. watermelon, honeydew) |
|  |  | Orange |
| **Drinks** |  | Peach or plum |
| Water |  | Tropical fruits (e.g. mango, guava) |
| Malt beverages (e.g. Milo®, Ovaltine®) |  | Dried fruits (e.g. raisins, dried apricot) |
| Fruit juice |  |  |
| Soft drinks (e.g. Coca-Cola®, Sprite®) |  | **Supplements** |
| ‘Diet’, ‘Zero’ or ‘Light’ soft drinks |  | Multivitamins |
| Other sweet drinks (e.g. Ribena®, Ice Lemon Tea) |  | Vitamin C |
| Sports drinks (e.g. 100 Plus®, H-Two-O®) |  | Fish oil |
| Cultured drinks (e.g. Yakult®, Vitagen®) |  | Probiotics |
| Soya milk |  |  |
| Coffee or Tea |  |  |
